# Characterization of Nucleosides and Nucleobases in Natural *Cordyceps* by HILIC–ESI/TOF/MS and HILIC–ESI/MS

**DOI:** 10.3390/molecules18089755

**Published:** 2013-08-15

**Authors:** Heng-Qiang Zhao, Xiao Wang, Hong-Mei Li, Bin Yang, Hong-Jun Yang, Luqi Huang

**Affiliations:** 1 Shandong Analysis and Test Center, Shandong Academy of Sciences, Jinan 250014, Shangdong, China; 2 National Resource Center for Chinese Materia Medica, China Academy of Chinese Medical Sciences, Beijing 100700, China; 3 Institute of Chinese Materia Medica, China Academy of Chinese Medical Sciences, Beijing 100700, China

**Keywords:** *Cordyceps*, nucleosides and nucleobases, hydrophilic interaction chromatography, electrospray ionization time-of-flight mass spectrometry, electrospray ionization tandem mass spectrometry

## Abstract

A method combining hydrophilic interaction chromatography (HILIC) and electrospray ionization mass spectrometry (ESI-MS) was developed for the characterization and determination of natural *Cordyceps*. Separation was achieved on a Waters Xbridge Amide column with gradient elution. Identification of 15 target nucleosides and nucleobases was based on retention time, UV spectra and mass measurements of the protonated molecules ([M+H]^+^) and main fragment ions (ESI-TOF/MS). Eight non-target compounds were tentatively identified by ESI-TOF/MS. The 15 target compounds were quantified by HILIC-ESI-MS/MS using time-programmed selective ion monitoring or multiple reaction monitoring in positive-ion mode under optimized mass conditions. This technique showed good linearity, repeatability and recovery. This approach was also successfully implemented in the analysis of nucleosides and nucleobases in 12 batches of natural *Cordyceps* samples that were collected from different regions in China. The developed HILIC-ESI-MS method exhibited clear advantages in identifying and determining highly polar bioactive components in *Cordyceps*, as well as their quality control.

## 1. Introduction

*Cordyceps*, found distributed mostly in the Gansu, Qinghai, Sichuan, Tibet and Yunnan provinces of China [1], has been widely used in Traditional Chinese Medicine (TCM) [1]. *Cordyceps* extracts have been shown to exhibit multiple pharmacological activities, such as anti-inflammatory and humoral-immunity suppressive properties [2,3,4]. Chemical studies have revealed that *Cordyceps* contains various components such as nucleosides and their bases, carbohydrates and sterols [5].Nucleosides and their bases are currently regarded as the bioactive components in *Cordyceps* [6,7]. Recent studies have indicated that nucleosides and nucleobases are involved in the regulation and modulation of human physiological processes [8,9]. Nucleosides and nucleobases have also been shown to exhibit various bioactivities [10,11], such as anti-platelet aggregation as well as anti-arrhythmic and anti-seizure effects [12,13,14]. Therefore, a specific and sensitive method to analyze these compounds is essential for the quality control of *Cordyceps*.

Various methods have been developed to detect nucleosides and nucleobases in *Cordyceps*, including reverse-phase high-performance liquid chromatography (RP–HPLC) [15,16], ultra-performance liquid chromatography (UPLC) [17], ion-pair reverse-phase chromatography (IP-RPC) [18,19], liquid chromatography–mass spectrometry (LC-MS) [20,21], capillary electrophoresis (CE) [22,23] and capillary electrophoresis–mass spectrometry (CE-MS) [24]. However, these methods have shown several disadvantages such as low separation efficiency for polar compounds, low sensitivity and uncertainty for non-target compounds without reference standards. Thus, these techniques cannot be used to characterize and quantitatively analyze multiple nucleosides and nucleobases in the extracts of *Cordyceps*, which exhibit high polarity and have complex matrices.

The HILIC method for the separation of polar compounds was developed by Alpert in 1990 [25]. In contrast to RP–HPLC, a hydrophilic polar stationary phase was used in HILIC, showing good reservation and separation efficiency for polar compounds. HILIC has been used in the analysis of several polar compounds in TCMs such as *Ligusticum chuanxiong* [26] and several medicinal marine organisms [27]. However, the possibility of simultaneously separating the 15 nucleosides, nucleobases and other components in the *Cordyceps* samples by using HILIC remains unknown.

The present study is the first to use HILIC to separate the nucleosides and their bases in *Cordyceps* samples. HILIC with diode-array detection and electrospray time-of-flight mass spectrometry was employed to confirm the target and the non-target compounds in the HILIC profile by comparing their UV spectrum and mass measurements with those of the reference standards or the literature. HILIC coupled with electrospray ionization tandem mass spectrometry (HILIC-ESI-MS/MS), which is more sensitive compared with routine HPLC [28], was then used for the simultaneous determination of multiple nucleosides and nucleobases for 12 natural *Cordyceps* samples from different cultivation regions. 

## 2. Results and Discussion

### 2.1. Optimization of HILIC-ESI-MS/MS Conditions

**Figure 1:**
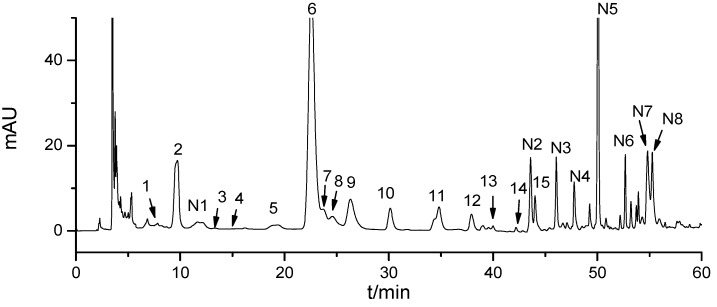
HILIC-DAD chromatograms of *Cordyceps* extract (**1**) Thymine; (**2**) Uracil; (**3**)Thymidine; (**4**) 2'-Deoxyuridine; (**5**) Cordycepin; (**6**) Uridine; (**7**) Hypoxanthine; (**8**) Adenine; (**9**) Adenosine; (**10**)Xanthine; (**11**) Inosine; (**12**) Cytosine; (**13**) Guanine; (**14**) Cytidine; (**15**) Guanosine;(N1-N8: non-target compounds).

In this study, a HILIC column was used to separate the high-polarity components in *Cordyceps* because of its poor retention on the reverse-phase column [29]. Chromatographic conditions were optimized using both nucleoside and nucleobase standards and a real natural *Cordyceps* sample. Firstly, three types of HILIC column, a Waters Xbridge^TM^ BEH Amide column (250 × 4.6 mm I.D., 3.5 μm), Waters Xbridge^TM^ HILIC column (150 × 2.1 mm I.D., 3.5 μm) and a Merck ZIC^®^ HILIC column (150 × 2.1 mm I.D., 3.5 μm) were compared. The results indicated that the former had better resolution for these hydrophilic components than the other two with the same mobile phase. Acetonitrile and water were selected for the mobile phase in the HILIC method based on a previous study [25]. Considering the complexity of the *Cordyceps* ingredients, gradient elution was selected to improve separation in the subsequent study. Besides, different mobile phase additives were compared for good resolution, peak shapes and compatibility with the ESI source. The result showed that the use of 0.2% acetic acid and 10 mmol/L ammonium acetate as mobile phase additives could provide much improved resolution and good peak shapes for these compounds. Figure 1 shows the HILIC chromatogram for the 60% (v/v) aqueous methanol extract of *Cordyceps*.

The mass spectrometry conditions were optimized to quantify 15 nucleosides and nucleobases, as well as to achieve maximum sensitivity. Preliminary direct infusion studies on the 15 standards confirmed that the positive ion mode was more sensitive. MS parameters such as nebulizer gas pressure, dry gas flow rate and dry temperature were optimized by injecting a mixture of standards. However, the results indicated that they had little effect on the sensitivity of the protonated molecules. Additionally, in tandem mass spectrometry, parameters such as cone voltage and collision energy were also adjusted for the most abundant product ion by using the fluxes and concentrations mentioned above. In this study, different collision energies determined by optimization experiments were applied to the 15 nucleosides and nucleobases. In addition, [M+H]^+^ was used as a precursor ion for most compounds. However, [M+Na]^+^ was selected for 2'-deoxyuridine. MRM was used to quantify the nucleoside, thereby minimizing background noise and improving sensitivity. However, the abundance of product ions for nucleobases was too low for detection using MRM; hence, SIM was used for the quantitative determination of these nucleobases. Table 1 shows the optimum parameters for MS/MS. 

**Table 1:** ESI-MS and ESI-MS/MS ions of nucleosides and nucleobases in *Cordyceps*.

Peak No.	Compounds	Precursor ions (*m/z*)	Product ions (*m/z*)	Monitoring mode	Collision energy(V)
1	Thymine	127.1 [M+H]^+^		SIM	-
2	Uracil	113.1 [M+H]^+^		SIM	-
3	Thymidine	243.1 [M+H]^+^	127.2 [M−116+H]^+^	MRM	14
4	2'-Deoxyuridine	250.9 [M+Na]^+^		SIM	-
5	Cordycepin	252.2 [M+H]^+^	136.1 [M−116+H]^+^	MRM	20
6	Uridine	245 [M+H]^+^	113 [M−132+H]^+^	MRM	8
7	Hypoxanthine	137 [M+H]^+^		SIM	-
8	Adenine	136.1 [M+H]^+^		SIM	-
9	Adenosine	268.2 [M+H]^+^	136.1 [M−132+H]^+^	MRM	20
10	Xanthine	153.1 [M+H]^+^		SIM	-
11	Inosine	269 [M+H]^+^	137.1 [M−132+H]^+^	MRM	22
12	Cytosine	112.1 [M+H]^+^		SIM	-
13	Guanine	152 [M+H]^+^		SIM	-
14	Cytidine	244.1 [M+H]^+^	112.1 [M−132+H]^+^	MRM	16
15	Guanosine	284 [M+H]^+^	152 [M−132+H]^+^	MRM	14

### 2.2. Qualitative Analysis for Cordyceps Extracts by HILIC-DAD-ESI-TOF/MS

**Figure 2:**
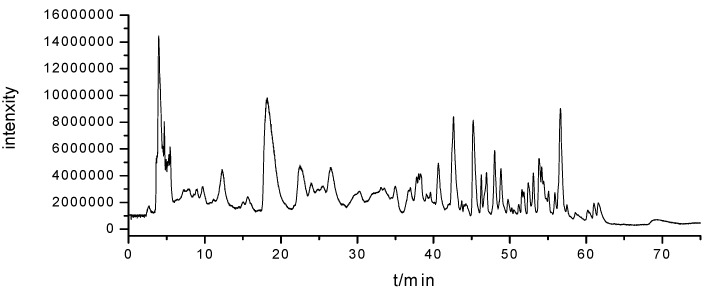
HILIC-ESI/TOF/MS chromatograms for *Cordyceps* extract.

The crude extract of *Cordyceps* was analyzed by HILIC-DAD-ESI-TOF/MS under optimized conditions, and the total ion chromatogram (TIC) of the *Cordyceps* sample is shown in Figure 2. It’s difficult to distinguish the peaks of the *Cordyceps* sample based on the TIC. However, 15 target nucleosides and nucleobases (peaks 1 to 15 in Figure 1) were unambiguously identified by their retention time, UV spectrum and accurate mass measurements in natural *Cordyceps*. Second, the molecular formula information of eight non-target compounds were also provided by ESI–TOF/MS. Table 2 lists 15 compounds found in the *Cordyceps* extract.

**Table 2:** HILIC-DAD-ESI-TOF/MS measurements for the extract of *Cordyceps*.

Peak No.	Retention time (min)	Compounds	Proposal ions	Elemental composition	Theoretical value *m*/*z*	Experimental *m*/*z*	Error (ppm)	UV λ_max _(nm)
1	8.1	Thymine	[M+H]^+^	C_5_H_7_N_2_O_2_	127.0502	127.0504	1.5742	260
2	9.8	Uracil	[M+H]^+^	C_4_H_5_N_2_O_2_	113.0346	113.0348	1.7694	262
3	13.8	Thymidine	[M+H]^+^	C_10_H_15_N_2_O_5_	243.0976	243.0979	1.2341	263
			[M−C_5_H_8_O_3_+H]^+^	C_5_H_7_N_2_O_2_	127.0502	127.0500	−1.5742	
4	15.7	2'-Deoxyadenosine	[M+H]^+^	C_10_H_14_N_5_O_3_	252.1091	252.1088	−1.9000	263
			[M+Na]^+^	C_10_H_13_N_5_O_3_Na	274.0911	274.0915	1.4593	
			[M−C_5_H_8_O_3_+H]^+^	C_5_H_6_N_5_	136.0618	136.0621	2.2049	
5	19.3	Cordycepin	[M+H]^+^	C_10_H_14_N_5_O_3_	252.1091	252.1096	1.9833	265
6	23.4	Uridine	[M+H]^+^	C_9_H_13_N_2_O_6_	245.0768	245.0771	1.2241	260
			[M−C_5_H_8_O_4_+H]^+^	C_4_H_5_N_2_O_2_	113.0346	113.0350	3.5387	
7	24.2	Hypoxanthine	[M+H]^+^	C_5_H_5_N_4_O	137.0458	137.0460	1.4594	257
			[2M+H]+	C_10_H_9_N_8_O_2_	273.0843	273.0841	−0.7324	
8	24.9	Adenine	[M+H]+	C_5_H_6_N_5_	136.0618	136.0621	2.2049	260
9	26.7	Adenosine	[M+H]+	C_10_H_14_N_5_O_4_	268.1040	268.1043	1.1190	262
			[M-C5H8O4+H]+	C_5_H_6_N_5_	136.0618	136.0622	2.9398	
10	30.4	Xanthine	[M+H]+	C_5_H_5_N_4_O_2_	153.0407	153.0405	−1.3068	262
11	35.2	Inosine	[M+H]+	C_10_H_13_N_4_O_5_	269.0881	269.0884	1.1149	263
			[M−C5H8O4+H]+	C_5_H_5_N_4_O	137.0458	137.0455	−2.1890	
12	38.1	Cytosine	[M+H]+	C_4_H_6_N_3_O	112.0505	112.0507	1.7849	257
			[2M+H]+	C_8_H_11_N_6_O_2_	223.0938	223.0941	1.3447	
13	40.2	Guanine	[M+H]+	C_5_H_6_N_5_O	152.0567	152.0571	2.6306	257
			[2M+H]+	C_10_H_11_N_10_O_2_	303.1061	303.1064	0.9898	
14	42.2	Cytidine	[M+H]+	C_9_H_14_N_3_O_5_	244.0928	244.0931	1.2290	262
			[M−C5H8O4+H]+	C_4_H_6_N_3_O	112.0505	112.0507	1.7849	
15	44.1	Guanosine	[M+H]+	C_10_H_14_N_5_O_5_	284.0990	284.0994	1.4080	260
			[M−C5H8O4+H]+	C_5_H_6_N_5_O	152.0567	152.0570	1.9729	
N1	12.1	Unknown	[M+H]+	C_17_H_23_N_10_O_8_	495.1695	495.1687	−1.6156	
N2	43.6	Unknown	[M+H]+	C_11_H_21_N_2_O_3_	229.1547	229.1553	2.6183	
N3	46.2	Unknown	[M+H]+	C_13_H_23_N_2_O_6_	303.1551	303.1549	−0.6597	
			[M+K]+	C_13_H_22_N_2_O_6_K	341.1109	341.1112	0.8795	
			[2M+Na]+	C_26_H_44_N_4_O_12_Na	627.2848	627.2852	0.6377	
N4	47.8	Unknown	[M+H]+	C_11_H_21_N_2_O_5_	261.1445	261.1441	1.5317	
N5	50.1	Unknown	[M+H]+	C_12_H_12_N_3_O_2_	230.0924	230.0929	2.1730	
			[M+Na]+	C_12_H_11_N_3_O_2_ Na	252.0743	252.0749	2.3803	
N6	52.3	Unknown	[M+H]+	C_7_H_16_NO_3_	162.1125	162.1121	−2.4674	
			[M+Na]+	C_7_H_15_NO_3_Na	184.0944	184.0939	2.7160	
N7	54.9	Unknown	[M+H]+	C_11_H_12_N_7_O	258.1098	258.1101	1.1623	
			[M+Na]+	C_11_H_11_N_7_ONa	280.0917	280.0922	1.7851	
N8	55.3	Unknown	[M+H]^+^	C_21_H_21_O_7_	385.1282	385.1286	1.0386	

**Figure 3:**
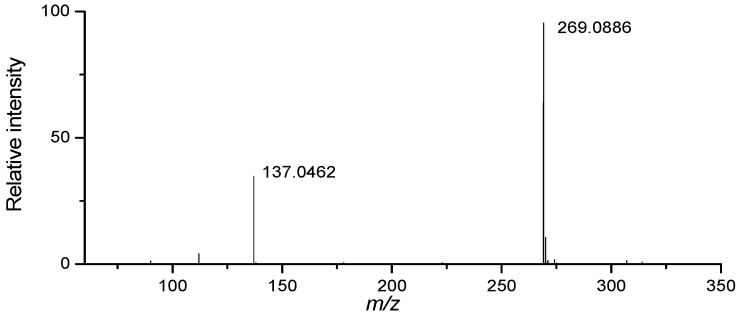
MS spectrum of peak 11.

For example, peak 11 (35.2 min) in the chromatograms in Figure 1 is identified by retention time, UV spectra and ESI–TOF/MS spectrum, as shown in Figure 3. First, it exhibits a maximum absorbance wavelength (λ_max_) of 265 nm, which characterizes nucleosides and nucleobases [24]. The exact masses of peak 11 are *m/z* 269.0886 and *m/z* 137.0462, as indicated in Figure 3. These measurements resulted from the [M+H]^+^ and the [M-C_5_H_8_O_4_+H]^+^ ions, which were consistent with the elemental compositions of C_10_H_13_N_4_O_5 _and C_5_H_5_N_4_O, respectively. After comparison of its retention time, UV spectra and MS spectrum with a reference standard, it was identified as inosine.

### 2.3. Validation of the Method

The linearity and calibration curves of 15 analytes were determined using the developed HILIC-MS/MS technique. All correlation coefficients obtained are higher than 0.9965, as presented in Table 3. Most linear dynamic ranges of the calibration curves for HILIC-MS/MS determination exceed two orders of magnitude. The limit of detection and the limit of quantification were defined at a signal-to-noise ratio of approximately 3 and 10, respectively. The detection limits of HILIC–MS/MS techniques range from 0.15 ng/mL to 10.00 ng/mL for the 15 nucleosides and nucleobases. This finding indicates that the developed method exhibits the same sensitivity as that in a previous report using HPLC–ESI–MS/MS [28] and a higher sensitivity than those reported using IP-RPLC-MS [19].

**Table 3:** Determination coefficientsof 15 nucleosides and nucleobases determined by HILIC-ESI-MS/MS assay.

Peak No.	Compounds	Regression equation	R^2^	Linear range (µg/mL)	LOD (ng/mL)	LOQ (ng/mL)
1	Thymine	y = 0.1312 x − 0.0034	0.9974	0.050–5.000	2.50	8.33
2	Uracil	y = 0.0491 x + 0.0005	0.9976	0.010–1.000	2.14	7.14
3	Thymidine	y = 0.0241 x + 0.0002	0.9999	0.050–5.000	10.00	33.33
4	2'-Deoxyuridine	y = 0.1368 x + 0.0011	0.9999	0.050–1.000	3.75	12.50
5	Cordycepin	y = 4.2277 x + 0.0670	0.9980	0.005–1.000	0.21	0.71
6	Uridine	y = 0.2125 x + 0.0008	0.9999	0.010–1.000	1.50	5.00
7	Hypoxanthine	y = 0.5223 x + 0.0220	0.9992	0.005–5.000	0.38	1.25
8	Adenine	y = 4.7164 x + 0.0783	0.9982	0.001–1.000	0.15	0.50
9	Adenosine	y = 2.0966 x + 0.1344	0.9980	0.010–5.000	0.60	2.00
10	Xanthine	y = 0.0752 x + 0.0008	0.9999	0.050–1.000	5.00	16.67
11	Inosine	y = 0.6156 x + 0.0717	0.9965	0.010–5.000	1.50	5.00
12	Cytosine	y = 1.7112 x − 0.0082	0.9993	0.010–1.000	0.86	2.86
13	Guanine	y = 0.2710 x + 0.0013	0.9997	0.010–5.000	2.50	8.33
14	Cytidine	y = 0.0829 x − 0.0001	0.9999	0.020–5.000	5.13	17.10
15	Guanosine	y = 0.0395 x + 0.0004	0.9974	0.050–10.000	7.50	25.00

Sample stability was tested by periodically analyzing a sample solution maintained at room temperature over varying periods (0, 2, 4, 6, 12 and 24 h). The storage stability of the measurements for the 15 nucleosides and nucleobases ranges from 3.25% to 5.69% (RSD, n = 6) for HILIC–MS/MS determination, as indicated in Table 4. Intra- and inter-day variations were chosen to determine the precision of the method. For the intra-day variability test, six replicate samples prepared independently were analyzed within one day. For the inter-day variability test, the replicate samples were analyzed in duplicate for three consecutive days. Total intra- and inter-day variations (RSD, n = 6) for the 15 analytes were less than 5.23% and 6.73%, respectively (Table 4). As shown in Table 4, the recoveries range from 83.6% to 117.3%, demonstrating the good recovery possible by using the method.

**Table 4:** Intra- and inter-day precision, stability and recovery for the 15 investigated compounds.

Peak No.	Compounds	Precision (RSD, %; n = 6)		Stability (R.S.D, %)	Recovery (n = 3, %)
		Intra-day	Inter-day		
1	Thymine	3.65	4.42	4.27	93.2
2	Uracil	3.71	3.29	3.95	87.5
3	Thymidine	2.89	4.61	4.11	96.7
4	2'-Deoxyuridine	2.45	5.27	4.38	105.3
5	Cordycepin	3.79	4.75	5.01	115.2
6	Uridine	2.95	3.31	3.85	92.1
7	Hypoxanthine	3.18	6.27	4.18	114.5
8	Adenine	3.60	4.59	3.89	107.3
9	Adenosine	4.58	5.33	4.53	84.6
10	Xanthine	4.21	5.72	5.27	105.7
11	Inosine	5.23	6.73	5.69	116.5
12	Cytosine	2.79	4.54	3.25	93.9
13	Guanine	4.17	5.18	4.93	83.6
14	Cytidine	4.25	4.65	5.31	117.3
15	Guanosine	4.83	5.89	4.57	92.1

**Table 5:** Contents ^a^ (μg/g) of 15 nucleosides and nucleobases in 12 batches of *Cordyceps* samples (n = 3).

Peak No.	Compounds	1#	2#	3#	4#	5#	6#	7#	8#	9#	10#	11#	12#
1	Thymine	1.88	2.42	1.54	5.16	5.27	5.89	4.43	2.31	16.10	5.39	2.26	3.49
2	Uracil	1.47	3.78	2.80	43.09	37.98	35.69	17.37	2.46	116.53	116.21	2.83	2.53
3	Thymidine	2.97	6.24	229.75	222.42	154.24	192.66	163.48	127.82	170.05	148.97	113.54	209.86
4	2'-Deoxyuridine	0.98	+ ^b^	+	3.19	3.23	3.54	3.82	2.42	2.58	4.86	2.09	1.49
5	Cordycepin	0.06	0.58	0.29	0.14	12.68	1.61	0.22	0.29	1.20	0.61	0.09	0.56
6	Uridine	161.30	39.49	190.09	190.99	273.30	119.11	160.69	59.44	22.85	23.68	72.08	124.01
7	Hypoxanthine	22.14	24.72	29.98	461.72	259.85	352.64	323.52	20.60	347.87	324.04	18.02	133.65
8	Adenine	23.24	46.95	59.45	31.16	33.99	15.58	29.59	29.33	22.90	16.63	29.21	37.24
9	Adenosine	123.77	393.74	480.25	147.87	157.59	93.12	151.30	354.05	92.37	46.40	250.47	203.09
10	Xanthine	2.09	+	+	121.19	102.46	194.46	172.88	0.78	638.78	747.31	1.28	5.99
11	Inosine	13.33	143.01	187.69	112.88	145.24	187.78	148.75	73.39	71.35	59.03	50.99	103.42
12	Cytosine	+	10.27	11.08	24.40	16.22	12.76	14.42	5.38	+	7.28	+	+
13	Guanine	1.05	0.86	0.50	33.01	13.39	7.82	12.85	4.93	18.82	60.38	2.42	8.08
14	Cytidine	2.06	23.90	4.97	2.88	+	+	4.76	6.89	10.52	5.70	6.74	+
15	Guanosine	32.10	34.69	146.42	123.79	123.42	139.01	128.67	54.073	49.67	56.35	41.25	80.16
total		388.43	730.64	1344.80	1523.89	1338.85	1361.69	1336.75	744.16	1581.59	1622.84	593.27	913.58

^a^ Average of triplicates (R.S.D.s <7%); ^b^ +Under the limits of quantification.

### 2.4. Quantitative Analysis of Investigated Compounds in Natural Cordyceps by HILIC-ESI/MS/MS

The newly developed HILIC-ESI-MS/MS method was applied for the simultaneous determination of 15 nucleosides and nucleobases (thymine, uracil, thymidine, 2'-deoxyuridine, cordycepin, uridine, hypoxanthine, adenine, adenosine, xanthine, inosine, cytosine, guanine, cytidine and guanosine) of 12 batches of *Cordyceps* samples that were collected from different regions in China. Table 5 summarizes the content of the investigated compounds in *Cordyceps*.

The results in Table 5 indicate that almost all *Cordyceps* samples are rich in nucleosides and nucleobases. The total content of the 15 nucleosides and nucleobases range from 388.43 μg/g to 1622.84 μg/g. The No. 10 sample from Suohumari Town, Qinghai Province has the highest content. Thymidine, uridine and adenine are rich in 12 natural Cordyceps samples. In addition, among the 12 samples, the No. 5 sample from Xiamagong Town, Qinghai Province exhibits the highest cordycepin content. Besides, the result also indicated that there were differences between the nucleosides and nucleobases contents of *Cordyceps* from different sources. The samples from different sources have various chemical profiles of nucleosides and nucleobases, which could be selected as the chemical markers for the quality control of *Cordyceps*.

## 3. Experimental

### 3.1. Materials and Standards

HPLC-grade acetonitrile was obtained from Merck (Darmstadt, Germany). Acetic acid and ammonium acetate were purchased from Fluka (Buchs, Switzerland). All other reagents such as methanol were of analytical grade (Tianjin Chemical Factory, Tianjin, China). Water was prepared using a Millipore Milli Q-Plus system (Millipore, Bedford, MA, USA).

Twelve batches of natural *Cordyceps* samples were collected from different regions in China (Table 6). The species of these natural *Cordyceps* was identified by Prof. Luqi Huang (Institute of Chinese Material Medical, China Academy of Chinese Medical Sciences, Beijing, China). 

**Table 6:** Regions of the 12 batches of *Cordyceps* samples.

Sample	Region	Sample	Region
1	Dawu Town, Qinghai Province	7	Manzhang Town, Qinghai Province
2	Dawu Town, Qinghai Province	8	Duogongma Town, Qinghai Province
3	Xueshan Town, Qinghai Province	9	Baiyu Town, Qinghai Province
4	Xueshan Town, Qinghai Province	10	Suohumari Town, Qinghai Province
5	Xiamagong Town, Qinghai Province	11	Lajishankou, Qinghai province
6	Jianshe Town, Qinghai Province	12	Naqu, Tibet

**Figure 4:**
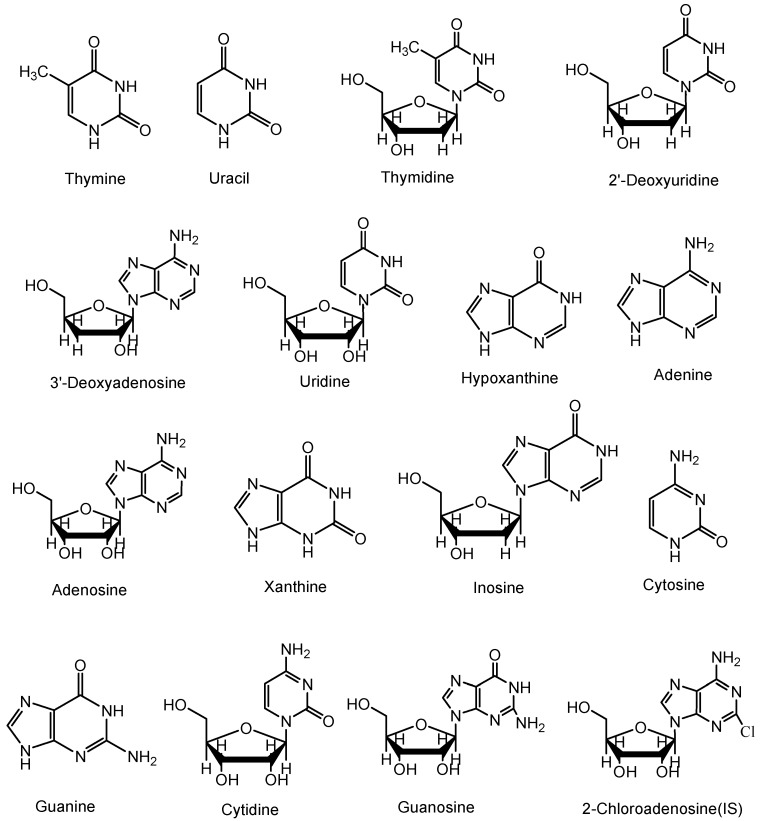
Chemical structures of nucleosides and nucleobases identified in *Cordyceps* extracts.

The standards of thymine, uracil, thymidine, 2'-deoxyuridine, cordycepin, uridine, hypoxanthine, adenine, adenosine, xanthine, inosine, cytosine, guanine, cytidine, guanosine and 2-chloroadenosine were purchased from Sigma-Aldrich (St. Louis, MO, USA). Figure 4 shows the chemical structures of these reference compounds. 

### 3.2. Sample Preparation

*Cordyceps* powder (0.3 g) was mixed with 60% (v/v) aqueous methanol (25 mL) in a glass tube with a stopper and then immersed in a conventional stainless steel ultrasonic cleaning bath. The average power used for extraction was 320 W. A single frequency of 40 kHz, as well as applied pulsed frequency, was used. Extraction was controlled at 20 °C. Samples were extracted in 15 min for three consecutive times. The extracts were combined and then filtered, and the filtrate was dried using a rotary evaporator at 55 °C. The residue was then dissolved in 5 mL of 90% (v/v) aqueous acetonitrile. The concentration of analytes was adjusted between linearity ranges according to the preliminary determination. 2-Chloroadenosine (1 µg/mL) was used as an internal standard for the quantitative determination of analytes by using a calibration curve. The solution was filtered through a 0.22 μm nylon filter membrane. A 20 μL aliquot of the filtrate was subsequently injected into the HPLC system.

### 3.3. HILIC-DAD

All separation processes were performed on an Agilent 1200 HPLC system (Agilent Technologies, Santa Clara, CA, USA) equipped with a quaternary solvent delivery system, a vacuum degasser, an autosampler, a column oven, and a diode-array detector connected to an Agilent ChemStation software program. A Waters Xbridge Amide column (250 × 4.6 mm I.D., 3.5 μm) was used. The mobile phase consists of A (0.2% acetic acid and 10 mM ammonium acetic) and B (acetonitrile), which are used in gradient elution: 0 min to 15 min, 95% to 95% B; 15 min to 30 min, 95% to 90% B; 30 min to 40 min, 90% to 80% B; 40 min to 45 min, 80% to 75% B. The flow rate of the mobile phase was 0.8 mL/min, and the injection volume was 20 μL. Elution was monitored at 260 nm by using a diode-array detector, and the column was maintained at 20 °C.

### 3.4. HILIC-ESI-TOF/MS Analysis

A G6520 Q-TOF mass spectrometer (Agilent Technologies) with an electrospray ion source was coupled with the Agilent 1200 HPLC system (Agilent Technologies) equipped with a Waters Xbridge Amide column (250 × 4.6 mm I.D., 3.5 μm) for the qualitative analysis of nucleosides and nucleobases in *Cordyceps*. Data were processed using MassHunter software (A02.02) provided with the Agilent TOF/MS. The mobile phase and the gradient elution program described in Section 3.3 were employed. The outlet of the flow cell was connected to a split valve to divert a flow of 0.4 mL/min to the ESI source. The mass spectrometer conditions were optimized for nucleosides and nucleobases detection, as follows: drying gas temperature, 350 °C; drying gas flow, 10.0 L/min; nebulizing gas pressure, 45 psi; fragmentor, 120 V; and capillary voltage, 4,500 V. The mass spectrometer was scanned from *m*/*z* 50 to *m/z* 1000 in a full scan mode. Reference solution was used, and the ion with *m*/*z* 121.050873 was selected for mass calibration to eliminate system bias.

### 3.5. HILIC-ESI-MS/MS Analysis

Quantitative analyses of nucleosides and nucleobases were performed on an Agilent 1200 HPLC system (Agilent Technologies) equipped with a Waters Xbridge Amide column (250 × 4.6 mm I.D., 3.5 μm) and coupled to an Agilent G6420A mass spectrometer (Agilent Technologies) triple quadrupole instrument. The mobile phase and the gradient elution program described in Section 2.3 were employed. The outlet of the flow cell was connected to a split valve to divert a flow of 0.4 mL·min^−1^ to the ESI source via a short fused silica tubing. Peaks were detected by a positive-ion mode MS and MS/MS. Mass spectrometry was conducted in the scan mode from m/z 50 to m/z 600. Selective ion monitoring (SIM) or multiple reaction monitoring (MRM) mode was used for quantitative analysis. The ESI-MS conditions were as follows: drying gas temperature, 350 °C; drying gas flow, 11.0 L/min; nebulizing gas pressure, 45 psi; fragmentation voltage, 120 V; and capillary voltage, 4500 V. 

### 3.6. Calibration Solutions

Standard stock solutions of all analytes, except for xanthine and guanine, were prepared by dissolving each compound in an aqueous methanol solution (20%, v/v) at 0.1 mg/mL and then stored at 4 °C. Xanthine and guanine (0.05 mg/mL) were prepared in aqueous acetic acid (2%, v/v) and then stored at 4 °C. The aforementioned standard solutions were prepared and diluted to appropriate concentrations with 90% (v/v) aqueous acetonitrile for the construction of calibration curves. Each calibration curve was generated by running samples at six different concentrations in triplicate. Relative peak areas were plotted against the concentrations of each analyte. The correlation coefficient was determined using a linear regression model.

## 4. Conclusions

In this study, a reliable technique for the simultaneous identification and determination of nucleosides and nucleobases in natural *Cordyceps* was developed by combination of HILIC–ESI–TOF/MS and HILIC–ESI–MS/MS approach. Fifteen nucleosides and nucleobases in 12 batches of natural *Cordyceps* were determined with the developed method. The results suggest that the developed method is specific and sensitive for the analytes examined, and can be used for the identification of non-target compounds. The analysis result showed that *Cordyceps* samples were rich in nucleosides and nucleobases, and they could be selected as markers for quality assessment of *Cordyceps*. Furthermore, the developed HILIC-ESI-MS method is a useful tool for the identification and determination of the highly polar bioactive components in *Cordyceps* as well as their quality control, and could also be used for the routine quantitation of nucleosides and nucleobases from other TCMs.
